# Macroeconomic fluctuations and individual use of psychotropic medications: evidence from Swedish administrative data

**DOI:** 10.1093/eurpub/ckac182

**Published:** 2023-01-09

**Authors:** Margareta Dackehag, Lina Maria Ellegård, Ulf-G Gerdtham, Therese Nilsson

**Affiliations:** Department of Economics, Lund University, Lund, Sweden; Department of Economics, Lund University, Lund, Sweden; Faculty of Business, Kristianstad University, Kristianstad, Sweden; Department of Economics, Lund University, Lund, Sweden; Department of Clinical Sciences (Malmö), Lund University, Malmö, Sweden; Department of Economics, Lund University, Lund, Sweden; Research Institute of Industrial Economics, Stockholm, Sweden

## Abstract

**Background:**

A growing literature finds that adult mental health worsens during economic downturns. Current insights on the relationship between macroeconomic fluctuations and psychotropic medication are based on self-reported information or aggregate measures on prescriptions. This study assesses the relationship between local macroeconomic conditions and individual use of psychotropic medication as reported in administrative registers.

**Methods:**

We use local information on unemployment linked to individual-level longitudinal data on detailed psychotropic drug consumption from administrative registers, for individuals in working age (20–65) in Sweden 2006–13. Any psychotropic medication uptake and the related number of redeemed prescriptions are the primary outcomes. Mortality is considered a secondary outcome.

**Results:**

Among young men (aged 20–44) and older women (aged 45–65), we find reduced use of psychotropic medication (2–4% compared to the mean) when the local labor market conditions deteriorate. The relationship is driven by reduced use of antidepressants. The same age-gender groups experience a significantly higher risk of mortality in bad times.

**Conclusions:**

This study shows that economic downturns may not only put strain on individuals’ mental health but also on their access to psychopharmaceutic treatments.

## Introduction

Mental health is an important issue on the global public health agenda. In 2019, almost 20% of the world’s population had a mental health disorder, and depression is one of the leading causes of disease burden.^1^ Poor mental health implies severe consequences for individuals, families and communities. (Estimates suggest that the annual societal costs of mental health disorders amount to $200 billion for the USA and EUR 600 billion for the EU.^2–4^)

Macroeconomic fluctuations may affect mental health. Job loss or workplace downsizing has adverse effects on individual mental health and wellbeing,^5^^,^^6^ and job search can be a salient stressor.^7^^,^^8^ Economic downturns may also increase job insecurity and feelings of powerlessness, anxiety and compassion for laid-off workers.^9^^,^^10^

A growing body of evidence suggests that mental health worsens during economic downturns,^11–15^ as indicated by suicide rates,^12^^,^^15–21^ emergency department visits due to overdoses^22^ and mental health hospitalizations.^23^ Yet, these outcomes do not capture milder instances of psychological distress. A complementary but limited strand of literature studies the use of psychotropic medication, which is relevant for broader shares of the population. In a systematic review, Silva *et al*.^24^ conclude that economic crises associate with higher self-reported use of prescription drugs, although sometimes only for certain groups.^25–27^

Studies on individual psychotropic medication generally rely on self-reported information,^25^^,^^27^ which may entail problems of misreporting and recall bias. Bharadwaj *et al*.^28^ show that individuals are significantly more prone to underreport mental illness compared to other health problems, suggesting that administrative data are particularly important for studies of mental health. To date, the small number of studies using administrative data to study the effects of macroeconomic conditions used aggregated data. (The focus of our study is the role of local macroeconomic conditions, i.e. the role of changes at the societal level, which may affect individuals indirectly, over and above the direct effect of own unemployment.^9^) Bradford and Lastrapes^29^ analyze regional monthly data from the USA from 1989 to 2009 and find a positive relationship between the unemployment rate and psychotropic drug prescriptions in one of the four regions. By examining the national-level data for member countries of the Organisation for Economic Co-operation and Development (OECD) from 1997 to 2017, Öztürk *et al*.^30^ note a positive relationship between yearly unemployment rates and average antidepressant use. Lundin and Hansson^31^ find a negative relationship between the monthly unemployment rate and the total number of prescriptions of antidepressants in Stockholm, Sweden, 1998–2008.

No existing study of administrative data has had access to individual-level information. A model specification based on individual-level data corresponds more closely to the relationship of interest, i.e. the impact of macroeconomic variables on individuals’ health behavior.^32^ A longitudinal micro-level approach further avoids interpretation difficulties stemming from compositional differences. By contrast, a positive association at the macro-level may reflect that unemployment induces selective migration of relatively healthy individuals who search for jobs elsewhere.

The aim of this study is to overcome these challenges using longitudinal individual-level administrative data. The research question is whether fluctuations in the local unemployment rate associate with the use of antidepressants, anxiolytics, hypnotics and sedatives among a representative cohort of Swedish individuals in working ages in 2006–13. We also examine the relationship between local economic conditions and mortality. Based on the previous literature, we hypothesize that mental illness increases during economic downturns.

## Methods

### Data and measures

We study data from official registers. The Prescribed Drug Register (PDR) covers the date and substances of all prescribed drugs dispensed at Swedish pharmacies. The Death Cause Register (DC) includes information from death certificates. PDR and DC are held by the National Board of Health and Welfare. We also use data from the Longitudinal Integrated Database for Health Insurance and Labour Market Studies (LISA) operated by Statistics Sweden, which contains individual- and household-level information on background characteristics such as income and income sources, educational attainment, civil status and region of residence.

To define a study population, we use data from the Swedish Survey of Living Conditions (SILC/ULF), which is conducted annually by Statistics Sweden. Each wave of the survey targets a population-representative sample and a small rotating panel (repeated every fourth year). Our study population includes the 153 160 individuals in working ages (20–65 years) who were surveyed at least once in 1980–2013 and still alive by 2005.

We link the study population to the PDR, DC and LISA using unique personal identifiers. Thus, we constructed a panel dataset containing annual information from official registers.

The primary outcome variables measure the individuals’ use of psychotropic drugs. We study three different classes of drugs: antidepressants (ATC category N06A), anxiolytics (N05B), and hypnotics and sedatives (N05C). These are the most used psychotropic drugs among both men and women. (Since our focus is on major ATC groups, we do not use antipsychotics (N05A) as an outcome. In 2006–18, the average number of adult women using antidepressants (N06A) per 1000 inhabitants in Sweden was 105.4, to compare with 18.9 for N05A.) We measure medication use both at the extensive margin (dichotomous variable equal to one if the individual redeemed at least one prescription during a year) and the intensive margin (number of redeemed prescriptions during a year). In sub-analyses, we study each of the three drug classes separately. We also study all-cause mortality (dichotomous variable equal to one if the individual died during the year).

We measure the local economic conditions by the annual average regional unemployment rate according to the official unemployment statistics from Statistics Sweden. Supplementary section S.1 discusses why unemployment is a good measure of the economic business cycle. In a sensitivity test, we study the annual rates of notifications of dismissal, as reported by the Swedish Public Employment Service.

We link information about the local economic conditions to the panel dataset on medication use and mortality using information on the individuals’ region of residence on December 31 the preceding year.

### Estimation strategy

We specify a linear model as follows:

$$y_{\mathrm{ijt}}=\alpha +{\beta BC}_{jt}+X_{\mathrm{ijt}}\gamma +T_{jt}\delta +\mu_{j}+\theta_{t}+\varepsilon_{\mathrm{ijt}},$$

where $y_{\mathrm{ijt}}$ is the outcome variable in year *t* for individual *i* residing in region *j*. In sub-analyses, we break down the outcome variable by substance. In a secondary analysis, we study the probability of death in a given year, in which case $y_{\mathrm{ijt}}$ is an indicator variable for having died in year *t*.

${BC}_{jt}\;$ is our business cycle measure for region *j* in year *t*. The coefficient of main interest is *β*, which captures the impact of the business cycle measure. *X* is a vector of individual background variables including sex, civil status, foreign background, having children under 18 years of age, living in a rural municipality, educational attainment (dummy variables indicating at most primary, secondary or tertiary education), and labor market status (dummy variables indicating receipt of welfare benefits, early retirement, pension and sickness or disability benefit). We also control for disposable household income (excluding negative and zero incomes). *α* is a constant, *T* refers to a region-specific time trend, $\mu_{j}$ is a region fixed effect, $\theta_{t}$ is a vector of year fixed effects and $\varepsilon_{\mathrm{ijt}}$ an error term. The specification with fixed effects and regional trends implies that we study variation in the unemployment rate in each region that is not due to an underlying trend in the regional unemployment rate, and not due to temporary shocks that affected the whole country.

In baseline estimations, we estimate the parameters of the model using ordinary least squares (OLS). Because the unemployment rate is measured at the regional level, we cluster standard errors by region level to account for correlation between individuals in the same region as well as for correlation over time within regions. Due to the clustering, the effective number of observations used to estimate the standard errors is substantially smaller than the total number of observations in our sample (closer to 21 annually). We therefore use a significance level of 0.10.

We perform several robustness tests. Since the cluster-robust standard errors may be sensitive to the small number of clusters (21 regions), we estimate standard errors using a wild cluster bootstrap procedure.^33^ (Simulation evidence indicates that tests based on the wild cluster bootstrap perform well in settings with a small number of clusters.^34^^,^^35^) To account for the binary nature of some dependent variables, we also estimate a logistic model for these outcomes. Other robustness tests include replacing the unemployment rate with the notice of dismissal rate, excluding covariates and adding the squared unemployment rate to account for non-linear relationships.

## Results

### Descriptive statistics

Table 1 presents descriptive statistics for the full sample and by groups defined by gender and age (20–44 and 45–65 years). Annually, more than 16% of the sample used any psychotropic medication. Antidepressants were the most used substances (10%). Consumption was higher among older compared to younger individuals, and women used more psychotropic medication than men, irrespective of age. The annual mortality rate was on average 0.3%, with large differences between the age groups.

**Table 1.** Descriptive statistics

	All		Men (20–44 years)		Men (45–65 years)		Women (20–44 years)		Women (45–65 years)	
	Mean	SD	Mean	SD	Mean	SD	Mean	SD	Mean	SD
Any psychotropic medication	0.165	0.371	0.080	0.271	0.132	0.339	0.145	0.353	0.236	0.425
Any anxiolytics (N05B) medication	0.054	0.226	0.028	0.165	0.044	0.204	0.048	0.215	0.075	0.263
Any hypnotics and sedatives (N05C) medication	0.083	0.277	0.032	0.177	0.070	0.256	0.053	0.225	0.127	0.333
Any antidepressants (N06A) medication	0.100	0.300	0.054	0.225	0.072	0.259	0.107	0.310	0.140	0.347
Redeemed prescriptions	1.171	5.925	0.555	4.034	0.986	5.653	0.918	5.102	1.679	6.964
Redeemed anxiolytics (N05B) prescriptions	0.272	2.271	0.141	1.708	0.242	2.156	0.190	1.870	0.385	2.672
Redeemed hypnotics and sedatives (N05C) prescriptions	0.423	2.631	0.165	1.743	0.371	2.484	0.268	2.316	0.634	3.107
Redeemed antidepressants (N06A) prescriptions	0.476	2.531	0.249	1.764	0.373	2.508	0.460	2.153	0.661	2.909
Mortality	0.002	0.050	0.001	0.024	0.004	0.064	0.0003	0.018	0.003	0.051
Regional unemployment rate %	7.523	1.385	7.459	1.379	7.552	1.388	7.444	1.375	7.554	1.387
Regional notice of dismissal rate %	1.259	0.669	1.254	0.666	1.261	0.673	1.252	0.664	1.261	0.670

*N*	1 028 624		149 841		33 867		156 384		383 729	

*N*, number of person-year observations; SD, standard deviation.

**Figure 1:**
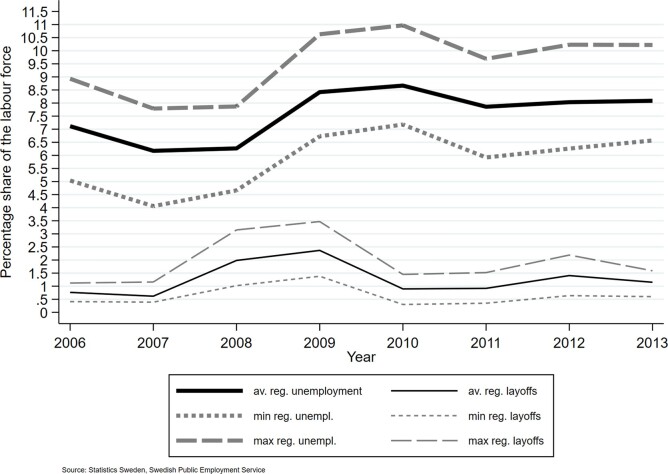
Regional unemployment and regional notice of dismissal rates 2006–13. The figure presents the average regional unemployment and notice of dismissal rates together with the corresponding annual maximum and minimum regional rates. The average notice of dismissal rate appears to move 1 year ahead of the regional unemployment rate. Otherwise, the two business cycle measures show similar patterns

The regional unemployment rate was around 7.5% on average over the study period. Figure 1 shows that the unemployment and dismissal rates develop similarly, although the dismissal rate moves 1 year ahead of the former. Supplementary figure SA1 shows that the unemployment rate varied substantially both over time and across regions. Supplementary figure SA2 shows a slightly positive trend in prescriptions of psychotropic medication 2006–13.

### Use of psychotropic medication

Table 2 shows the main results. For the study population as a whole (column 1), a worsening of the local macroeconomic conditions associates with lower psychotropic medication, both when examining any uptake (Panel A) and the number of redeemed prescriptions (Panel B). A one percentage point increase in the unemployment rate associates with a 0.11 percentage point (p.p) reduction in the probability to use psychotropic medication, and with a decrease of 0.02 redeemed prescriptions per year. Putting these estimates in relation to the mean values of the outcome variables, the estimates correspond to reductions of close to 0.7% and 2%. The independent variables explain 7–13% of the variation in the dependent variables, showing that most of the variation remains unexplained.

**Table 2.** Regional unemployment rate, psychotropic medication and mortality

	All	Men (20–44 years)	Men (45–65 years)	Women (20–44 years)	Women (45–65 years)
Panel A					
Any psychotropic medication	−0.00109	−0.00128	0.000221	0.00138	−0.00304
	(−0.00209, −0.0000993)	(−0.00360, 0.00104)	(−0.00139, 0.00183)	(−0.000617, 0.00338)	(−0.00461, −0.00147)
R2	0.131	0.114	0.100	0.121	0.116
*N*	1 026 074	149 753	337 279	156 331	382 711
Panel B					
Number of redeemed prescriptions	−0.0161	−0.0295	−0.00751	−0.00789	−0.0191
	(−0.0245, −0.00768)	(−0.0570, −0.00206)	(−0.0212, 0.00616)	(−0.0274, 0.0116)	(−0.0333, −0.00486)
R2	0.0758	0.0969	0.0682	0.0873	0.0710
*N*	1 026 074	149 753	337 279	156 331	382 711
Panel C					
Mortality	0.0000900	0.000336	−0.000265	0.0000822	0.000320
	(−0.0000360, 0.000216)	(0.0000564, 0.000616)	(−0.000626, 0.0000968)	(−0.0000529, 0.000217)	(0.0000647, 0.000576)
R2	0.00487	0.00143	0.00655	0.00127	0.00368
*N*	1 026 551	149 242	338 223	155 653	383 433

Estimates from ordinarly least squares models (Panels A–C). Regression results are based on different (sub)samples: men and women 20–65 years (All), men 20–44 years, men 45–65 years, women 20–44 years and women 45–65 years. The results show the estimated coefficients on the business cycle measure, the share of unemployed individuals in the regional labor force (15–74 years) in the current year. In the row below each estimated coefficient, the lower and upper bound of the 90% confidence intervals are found in parentheses. The dependent variable in Panel A is a dichotomous variable indicating any use of psychotropic medication (at least one drug belonging to any of the ATC categories N05B, N05C and N06A) per individual and year. The dependent variable in Panel B measures the number of redeemed prescriptions per individual and year. The dependent variable in Panel C is a dichotomous variable indicating death in the current year. All models control for region fixed effects, year fixed effects, region time trends and individual background variables (in Panel C the time-varying background variables are measured in the previous year).

Splitting the full sample into four groups defined by gender and age, we find that the main results are primarily driven by younger men (aged 20–44; column 2) and older women (aged 45–65; column 5). As the local unemployment rate increases by one p.p, the older women are 0.3 p.p less likely to use any psychotropic medication (1.3% compared to the group mean), and decrease the number of redeemed prescriptions by 1.1% on average. For young men, the estimate on the probability of use rate is negative but not statistically significant, while the association with the number of redeemed prescriptions corresponds to a decrease of over 5% of the group mean. For younger women and older men, the associations are never statistically significant.

Supplementary tables SA2–SA4 show that the above results mainly reflect a lower use of antidepressants and a reduced number of redeemed prescriptions of anxiolytics among younger men and older women. Notably, the reduction in the probability of using antidepressants is statistically significant for young men, indicating that the insignificant estimate in table 2 for this sub-group is due to the weaker associations for the other substances. The analysis by substance type also reveals a large and significant reduction in the number of redeemed prescriptions of anxiolytics for younger women.

### Mortality

Panel C of Table 2 shows estimates of the relationship between local macroeconomic fluctuations and mortality. The results for the whole sample are insignificant, but results by subgroup align with the findings for psychotropic medication: increases in unemployment rates associate with increases in mortality for younger men and older women. The associations amount to 0.03 p.p, or 30% compared to the (very low) mean for young men, and 0.03 p.p, corresponding to 10% compared to the mean, for older women.

Individuals who pass away in a given year have shorter time to redeem prescriptions, and therefore mechanically use less drugs than individuals who survive the whole year. Our results in the previous section do not reflect such a mechanical correlation, as we exclude the censored observation (i.e. from the year of death) for individuals who passed away.

### Robustness tests

As a first robustness test, we change our model specification and exclude all individual covariates. This exercise generates results very similar to the baseline (Supplementary table SA5; Model 1, Supplementary File).

We also use logistic regressions to estimate the relationship for the dichotomous dependent variables. Supplementary table SA6 shows that these results corroborate our baseline findings. (Logit models of business cycle fluctuations and mortality fail to converge.) We also examine whether the relationship of interest varies with the severity of local economic downturns, by including both the level and the square of the unemployment rate. The results do not indicate a non-linear relationship.

In line with baseline findings, the estimations using the notice of dismissal rate as the business cycle measure suggest that there is a negative and significant relationship between economic downturns and redeemed prescriptions for older women, driven by a reduced number of redeemed prescriptions of antidepressants and of hypnotics and sedatives (see Supplementary tables SA7–SA10). The results for young men are imprecisely estimated, but magnitudes are large. An increase by one p.p in the dismissal rate associates with a 6% decrease in the number of redeemed prescriptions.

Table 3 shows that the standard errors are only marginally affected when we use the bootstrap procedure instead of regular cluster-robust standard errors.

**Table 3.** Local macroeconomic conditions and psychotropic medication for men and women 20–65 years old—a bootstrap approach

	Regional unemployment rate	Regional notice of dismissal rate
Panel A		
Any psychotropic medication	−0.00109	−0.00183
Original CI	(−0.00209, −0.0000993)	(−0.00374, 0.0000822)
Bootstrapped CI	(−0.00223, 0.0000186)	(−0.00414, 0.000538)
Panel B		
Number of redeemed prescriptions	−0.0161	−0.0224
Original CI	(−0.0245, −0.00768	(−0.0380, −0.00672)
Bootstrapped CI	(−0.0244, −0.00671)	(−0.0394, −0.00250)

Estimates from ordinary least squares models (Panels A and B) based on bootstrapped standard errors. Regression results refer to men and women 20–65 years. The results show the estimated coefficients on the business cycle measure, the share of unemployed individuals in the regional labor force (15–74 years) in the current year (second column) and the share of individuals with notice of dismissal in the regional labor force (15–74 years) in the previous year (third column). In the row below each estimated coefficient, the lower and upper bound of the 90% confidence intervals are found in parentheses. In the next row, the lower and upper bound of the bootstrapped 90% confidence intervals are shown in parentheses. The dependent variable in Panel A is a dichotomous variable indicating any use of psychotropic medication (at least one drug belonging to any of the ATC categories N05B, N05C and N06A) per individual and year. The dependent variable in Panel B measures the number of redeemed prescriptions per individual and year. All models control for region fixed effects, year fixed effects, region time trends and individual background variables.

## Discussion

The existing literature on suicides and the business cycle suggest that mental health deteriorates during economic downturns. The use of psychotropic medications, which aim to mitigate the effects of mental health disorders, may therefore also display a cyclical pattern. Studies of self-reported individual psychotropic medication suggest that the increased prevalence of mental ill-health is coupled by increases in medication use, at least for certain groups (cf. Ref. ^28^). A positive relationship has also been found at the population level in parts of the USA^29^ and OECD countries.^30^ These results are consistent with a story in which the use of psychotropic medication first and foremost is a proxy for mental ill-health.

The present study using administrative individual-level data fails to support our main hypothesis that the use of psychotropic medication increases during economic downturns. Our results indicate the complete opposite: we find a negative association between the local unemployment rate and the use of psychotropic drugs. The relationship is driven by young men and older women. Interestingly, we find increased mortality in the same population groups, which is consistent with, if not direct evidence of, worsened mental health during economic downturns.

These findings can be reconciled by recognizing that the use of psychotropic medications does not only function as a proxy of mental health but may also reflect compensatory health behavior and access to drugs. It is outside the scope of this study to conduct a thorough investigation of the many potential mechanisms, but we discuss some possible explanations.

On the demand side, financial strain during economic downturns may reduce individuals’ ability to afford the co-payment for doctor visits or fill prescriptions. Analyzing regional-level statistics on primary care visits, we cannot rule out that the unemployment rate associates with either large decreases or increases in the number of visits per capita. (We merged annual regional data from www.kolada.se with our unemployment data to estimate these associations.) We also examine the relationship between unemployment rate and per capita visits to specialist psychiatric care at the regional level. While the point estimate for the local business cycle is negative, it is far from being significant. However, a previous study from Sweden found that individuals who recently became unemployed were more likely to abstain from seeking care despite a perceived care need.^36^ Such behavior could reduce the likelihood to initiate a new drug treatment. Furthermore, the patient co-payment for a 100-day treatment of antidepressant was around EUR 30 in our study period.^37^ This could be a significant outlay for individuals with low income (the average monthly net earnings for blue-collar workers in 2010 was around EUR 1600). (Even though there is an annual cap for patient drug co-payments set at EUR 240 in Sweden, the patient pays the full amount up to around EUR 100, and then a decreasing share of costs up to the EUR 240 cap. Drug costs above that cap are fully subsidized.)

Regardless of the individual’s employment status, increased worry about deteriorations in the economic conditions may lead to increased use of alcohol and illegal drugs. The use of other drugs would likely not make individuals cancel ongoing treatments but may reduce the propensity to initiate new treatment. In exploratory analyses relating local unemployment rates to a public health survey, we were unable to either confirm or dismiss cyclical patterns in the self-reported consumption of alcohol, tobacco and cannabis use nor when examining statistics on regional per capita sales of types of alcohol, but the issue ought to be examined using better data.

On the supply side, tighter healthcare budgets during downturns may reduce physicians’ propensity to prescribe medicine. During the study period, the Swedish regional health authorities announced budget cuts and made efforts to contain drug costs, due to expectations of lower tax revenues following the onset of the Great Recession.^38^ Looking at region-level statistics on the drug costs in primary care and psychiatric care and the total number of redeemed prescriptions (any drug), we find that the unemployment rate is negatively associated with psychiatric care drug costs, but not with primary care drug costs and total prescriptions. These findings do not provide strong evidence in favor of budget cuts as an explanation, although it should be acknowledged that these associations are driven by not only supply-side factors but also by demand-side factors.

Another potential reason for the discrepancy of our results and those in previous studies relates to the nature of the data. If individuals are more likely to understate, or less attentive to, their psychotropic medication in good times—e.g. because the stigma of mental ill-health is weaker in ‘objectively bad’ situations—then the pattern in analyses of self-reported data may be overstated due to systematic differences over the business cycle in misreporting or recall bias. While our data does not allow us to estimate the relative importance of misreporting, recall bias and context-specific circumstances, we can conclude that the previous evidence on a positive relationship between economic downturns and psychotropic medication is not generalizable to all settings. In this regard, it should be noted that our results are similar to those of a previous study from one Swedish region using aggregate data from 1998 to 2008.^31^ Since the late 1990s, Swedish local government and health authorities have been subject to a strict fiscal framework requiring budget balance.^39^ Possibly, the negative associations found in both our and the previous Swedish study reflect swift responses on the supply side, which may be absent in other settings with less fiscal discipline.

As we had access to data on all redeemed prescriptions of psychotropic medication, regardless of where in Sweden the dispensing pharmacy was located, our results cannot be explained by sample attrition due to movements between regions.

The study has several limitations. While our data include all dispenses of the selected psychotropic medications at Swedish pharmacies, the prevalence of dispensed drugs is not a perfect measure of the prevalence of mental ill-health, as it does not capture individuals with severe mental problems who abstain from seeking treatment. Further, our data only include the number of redeemed prescriptions, whereby we cannot disentangle the relative importance of changes in prescription patterns, nor study the daily defined doses. Finally, our statistical models study annual variations in economic conditions, and thus do not indicate how the relationship of interest develops in the long run.

To conclude, we document that individuals’ use of psychopharmaceutic drugs falls as local labor market conditions deteriorate. For the groups with the strongest reductions in using psychotropic medication, we also see increased mortality in subsequent years. Our findings are relevant for social policy in the domain of mental health, but also in view of the recent COVID-19 pandemic that generated a shock to many economies. Previous evidence on suicide, mental health-related hospitalizations and emergency visits suggest that scaling up treatment of depression and anxiety disorders could reduce the negative impact of downturns on mental health. Yet, our results, from a context with a large welfare state and a strong social security net, indicate that downturns by themselves pose a challenge to the ability to access and/or demand the treatments that would relieve mental problems. Increasing awareness about the potentially synchronized downturns in economic and mental conditions is a first step to help decision-makers formulate policies that may counteract the potentially detrimental consequences on mental ill-health.

## Supplementary Material

ckac182_Supplementary_Data

## Data Availability

The data underlying this article cannot be shared publicly due to the European and Swedish legislation surrounding the use of sensitive personal information. Economic downturns can put strain on individuals’ mental health and the public healthcare budget. We study the role of local economic conditions for individual use of psychotropic medications. Young men and older women use psychotropic medications, especially antidepressants, less during economic downturns. The same age-gender groups experience a higher risk of mortality.
