# Supplementary material for: Macroeconomic fluctuations and individual use of psychotropic medications: evidence from Swedish administrative data
Source: Eur J Public Health. 2023 Jan 9;33(1):93–8. doi: 10.1093/eurpub/ckac182 (PMC9898001; doi:10.1093/eurpub/ckac182)
Supplement: ckac182_Supplementary_Data [file ckac182_supplementary_data.docx]

**Supplementary material (for online publication)**

**Section S.1**

We use the regional annual unemployment rate from Statistics Sweden as the main explanatory variable to capture local business cycles. Unemployment is generally seen as a relevant proxy as labor market changes are very important in evaluating where in the business cycle an economy is, and there exists reliable and frequently updated data on unemployment for various geographical aggregations.

A majority of published article on the relationship between the recessions and health use information on unemployment, see e.g. Ruhm (2000), Neumayer (2004), Economou et al. (2008), Gerdtham and Ruhm (2006). As pointed out by Gerdtham and Johannesson (2005) potential disadvantages of using unemployment as a proxy of the business cycle is that unemployment statistics does not capture changes in the work force and does not include individuals targeted by labor market programs which we know tent to increase in economic downturns. Another potential weakness is that it may take some time from the point in time when the local economy changes until we see changes to unemployment. As a second measure to capture local business cycles we use the annual rate of the regional labor force notified of dismissal, as reported by the Swedish Public Employment Service. Notices of dismissal may function as an early signal of a worsening macroeconomic situation and may capture job insecurity, which has been shown to affect mental health (34).

**Section S.2**

**Figure A1. Fluctuation in regional unemployment rates 2006-2013**

**
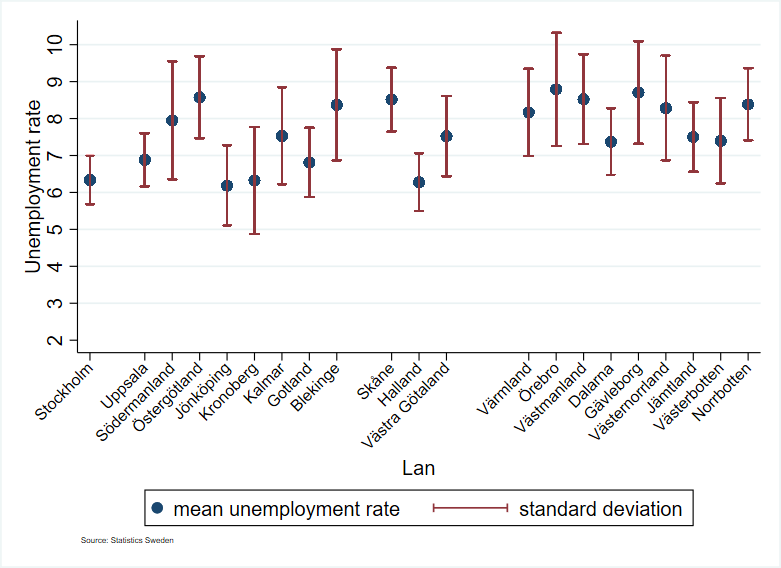
**

*Notes:* The figure illustrates the average unemployment rate in each region of Sweden 2006-2013. It also illustrates the standard deviation from the mean unemployment rate in each region over the same years.

**Figure A2. Psychotropic medication 2006-2013**


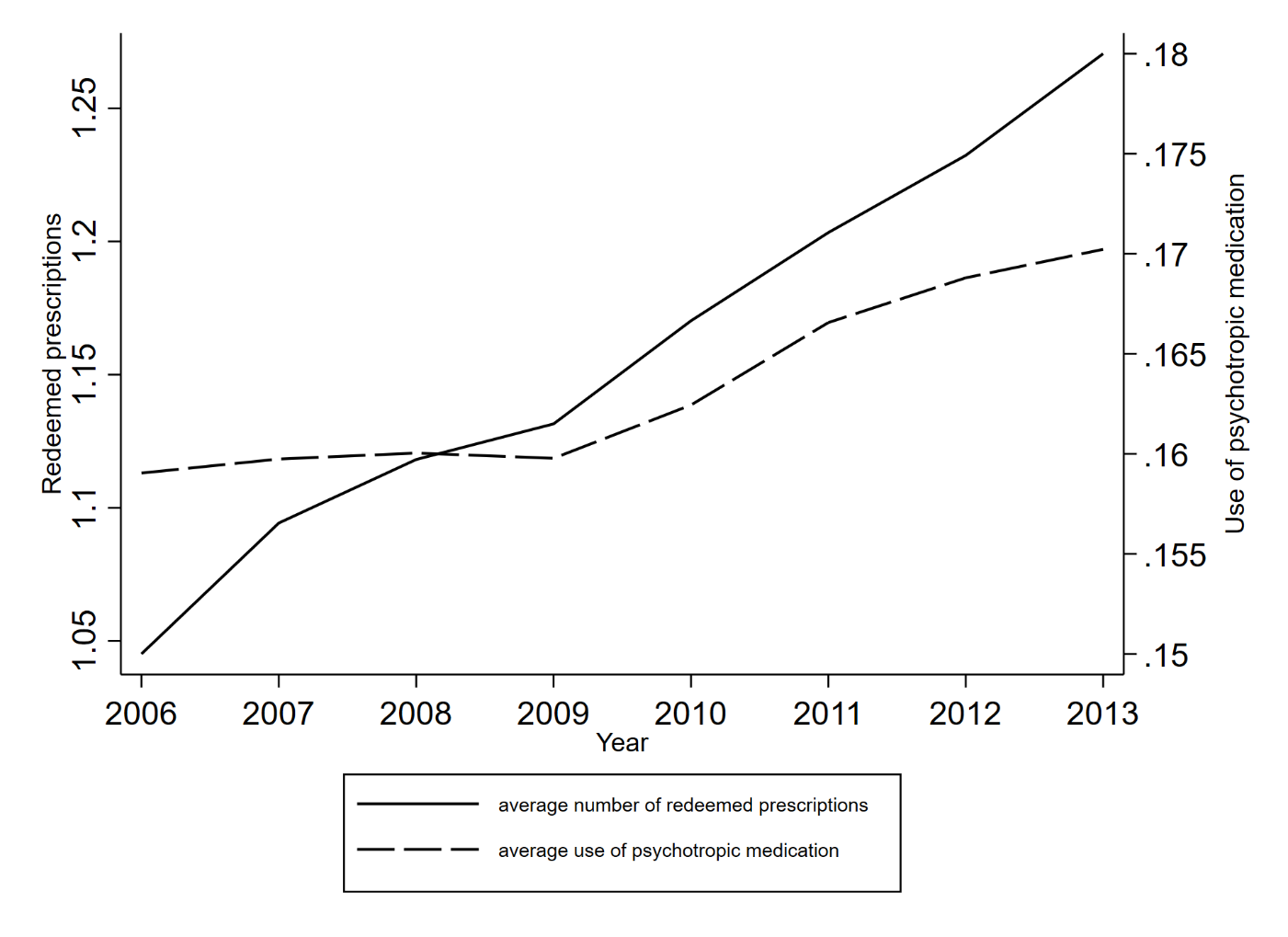


Note: Annual average use of psychotropic medication and annual average of redeemed psychotropic prescriptions. Psychotropic medication belonging to ATC group N05B, N05C and N06A. Source: Swedish Prescribed Drug register

**Table A1. Descriptive statistics of individual control variables**

**Table A2. Regional unemployment rate and psychotropic medication use by type of substance**

**Table A3. Regional unemployment rate and psychotropic medication use by type of substance**

**Table A4. Regional unemployment rate and psychotropic medication use by type of substance**

**Table A5. Regional unemployment rate, psychotropic medication use and mortality when adding covariates to the model - Men and women 20-65 years old**

**Table A6. Regional unemployment rate and psychotropic medication use – logistic regressions**

**Table A7. Regional notice of dismissal rate, psychotropic medication use and mortality**

**Table A8. Regional dismissal rate and psychotropic medication use by type of substance**

**Table A9. Regional dismissal rate and psychotropic medication use by type of substance**

**Table A10. Regional dismissal rate and psychotropic medication use by type of substance**
